# A machine learning computational approach for the mathematical anthrax disease system in animals

**DOI:** 10.1371/journal.pone.0320327

**Published:** 2025-04-01

**Authors:** Zulqurnain Sabir, Eman Simbawa

**Affiliations:** 1 Department of Computer Science and Mathematics, Lebanese American University, Beirut, Lebanon; 2 Department of Mathematics, Faculty of Science, King Abdulaziz University, Jeddah, Saudi Arabia; Satyawati College, University of Delhi, INDIA

## Abstract

**Objectives:**

The current research investigations present the numerical solutions of the anthrax disease system in animals by designing a machine learning stochastic procedure. The mathematical anthrax disease system in animals is classified into susceptible, infected, recovered and vaccinated.

**Method:**

A Runge-Kutta solver is applied to collect the dataset, which decreases the mean square error by dividing into training as 78%, testing 12% and verification 10%. The proposed stochastic computing technique is performed through the logistic sigmoid activation function, and a single hidden layer construction, twenty-seven numbers of neurons, and optimization through the Bayesian regularization for the mathematical anthrax disease system in animals.

**Finding:**

The designed procedure’s correctness is authenticated through the results overlapping and reducible absolute error, which are calculated around 10^-05^ to 10^-08^ for each case of the model. The best training performances are performed as 10^-10^ to 10^-12^ of the model. Moreover, the statistical performances in terms of regression coefficient, error histogram, and state transition values enhance the reliability of the proposed stochastic machine learning approach.

**Novelty:**

The designed scheme is not applied before to get the numerical results of the anthrax disease system in animals.

## 1. Introduction

Anthrax is a severe zoonotic illness, which is often recognized as a fatal one and affects the animals worldwide. It is produced through the bacterium Bacillus anthracis, which primarily distresses the herbivores, e.g., sheep, cattle, horses, and goats [1]. This illness spreads by the direct interaction with infected creatures, spores in soil, contaminated products, feed or water. These spores can continue practical for periods, which make anthrax as a persistent danger. The disease system based on the anthrax in animals shows numerous clinical procedures, e.g., gastrointestinal, cutaneous, septicemic anthrax, and pulmonary [2]. Cutaneous anthrax produces skin lesions, whereas digestive anthrax results from ingesting contaminated water or food. Pulmonary anthrax distresses the septicemic anthrax, and lungs, which pollutes the bloodstream. Symptoms comprise lethargy, fever, bloody discharge, appetite loss, swollen lymph nodes, and hard breathing. Prompt analysis done by clinical indications and laboratory testing is essential for operative action. Biosecurity and vaccination actions are life-threatening for avoiding the anthrax outbursts in the populations of animals. For the treatment of this disease, vaccines based live spore are generally used, while antibiotics, e.g., doxycycline and penicillin can be used to treat the diseased animals. Appropriate treatment and clearance of animal yields, disinfection, and quarantine measures are also helpful to prevent the spreading of this disease. To understand the anthrax epidemiology based on the endemic areas along with risk influences is used to notify the public health policies in order to lessen the bioterrorism intimidations, and zoonotic communication. The effective anthrax management in the creatures protects both human health and animal.

An extended area of mathematics involves the examination and exploration of diverse mathematical representations based on the natural phenomena. Using rational and computational methods, scientists examine the behavior of specified structures. This process shows the new fractional operatives, which has a significant role to model such natural procedure and phenomena. Currently, the fractional operator based Caputo–Fabrizio is implanted by a number of scholars to examine the current models [3–4]. There are various investigations that have been applied based on the fractional calculus [5–7]. The Bacillus Anthracis bacteria are thought to be the source of anthrax, a viral illness. Considering the category of zoonotic infections, anthrax disease impacts both people and wildlife populations [8]. Obviously soil contains the anthrax pathogen, which primarily affects the animals rather than carnivores. It is the most deadly transmissible illnesses in the entire globe, which causes a significant and uncontrollable death rate across certain animal groups, including pigs, cattle, sheep, goats, and horses [9–10]. Gutting et al. [11] stated the category of creatures, which can contract the Bacillus Anthracis pathogen in a number of ways, such as consuming contaminated grass or water, breathing in its microorganisms or coming into touch with diseased animals. Keeping in mind that infected carcasses of livestock can potentially contaminate the surroundings. Although anthrax spores may survive though grass or soil for extended periods of time in extremely adverse climates, these two environments are the most significant repositories of anthrax spores, which can spread the illness from one animal to another. Additionally, anthrax takes up to eight days to incubate when an animal dies, the clinical signs of the illness require time to appear in animals with the infection.

The current research presents the numerical performances of the anthrax disease system in animals by designing a machine learning stochastic procedure using the optimization of Bayesian regularization. The proposed artificial neural network (ANN) procedure along with Bayesian regularization has never been tested before to solve the mathematical anthrax disease system in animals. Recently, the stochastic numerical performances have been tested in a number of submissions. To mention some of them are fluid mathematical model [12], thermal explosion system [13], human movement model [14], mosquito spreading system [15], language learning model [16], hard water consumption with kidney model [17], Zika virus spreading model [18] and many more [19–25]. Based on these applications, the authors explored the numerical results of the mathematical anthrax disease model in animals by applying a neural network procedure of Bayesian regularization. Few novel topographies of current investigations are presented as:

- Finding the numerical performances of the nonlinear systems with conventional techniques is typically difficult. Therefore, the suggested optimization Bayesian regularization neural network approach successfully addresses these complicated problems.
- The numerical solution of the nonlinear system is obtainable effectively by implementing the proposed ANN based Bayesian regularization structure.
- A single hidden layer structure using the sigmoid activation function along with twenty-seven numbers of neurons is applied to solve the nonlinear model.
- The precision of the designed ANN based Bayesian regularization procedure is obtained through the matching of the results and reducible absolute error (AE).

The other paper parts are given as: Mathematical anthrax disease system in animals is shown in Section 2, a process of Bayesian regularization is presented in Section 3, and the numerical results are given in Section 4, however the conclusions are provided in section 5.

## 2. Mathematical anthrax disease system

This section shows the mathematical model, which is classified into four nonlinear classes, susceptible, infected, recovered and vaccinated. The nonlinear first order anthrax disease model in animals is expressed as [26]:


$$\{\begin{array}{l} \frac{dS(x)}{dx}=\omega -\delta I(x)S(x)-(\upsilon +\rho )S(x)+\varpi V(x)+\xi R(x),\\ \frac{dI(x)}{dx}=\delta I(x)S(x)-(\tau +\kappa +\rho )I(x),\\ \frac{dR(x)}{dx}=\kappa I(x)-(\xi +\rho )R(x),\\ \frac{dV(x)}{dx}=\upsilon S(x)-(\varpi +\rho )V(x). \end{array}$$ (1)

Subjected to the positive initial conditions (ICs). The human contribution based on the anthrax disease spreading in the animals is insignificant, and it has gotten a huge significance to transmit the illness in the population of animals. *S*(*x*) presents the susceptible using the animal numbers at risk based on the anthrax contagion, *I*(*x*) shows the infected based on the number of animals that have disease symptoms, *R*(*x*) is the recovered animal numbers, which have anthrax infection and attained chronological immunity, and the last class *V*(*x*) indicates the vaccinated animal numbers due to the attacks of anthrax disease. *ω* designates the rate of enrollment, *δ* is the rate of contact, *ρ* presents the natural rate of death, *υ* signifies the rate of vaccination, *ζ* is the waning rate of recovery, *ϖ* denotes the waning immunity rate based on vaccinated animals, *τ* is the rate of induced- disease death, while *κ* parameter shows the recovered animals rate.

## 3. Methodology

The proposed stochastic procedure is designed mathematically by taking 27 neurons and the logistic sigmoid function (LSF) in the hidden layer, which is shown as:


$$\left[\begin{array}{l} r_{1}\\ r_{2}\\ r_{3}\\ .\\ .\\ .\\ r_{27} \end{array}\right]=ϒ\left(\left[\begin{array}{l} w_{1,1}\\ w_{1,2}\\ w_{1,3}\\ .\\ .\\ .\\ w_{1,27} \end{array}\right][x]+\left[\begin{array}{l} b_{1,1}\\ b_{1,2}\\ b_{1,3}\\ \begin{array}{c} .\\ \begin{array}{l} .\\ . \end{array}\\ b_{1,27} \end{array} \end{array}\right]\right),$$ (2)

$$\left[\begin{array}{c} S(x)\\ I(x)\\ R(x)\\ V(x) \end{array}\right]=\left(\left(\begin{array}{ccccccc} \omega_{1,1} & \omega_{2,1} & \omega_{3,1} & . & . & . & \omega_{27,1}\\ \omega_{1,2} & \omega_{2,2} & \omega_{3,2} & . & . & . & \omega_{27,2}\\ \omega_{1,3} & \omega_{2,3} & \omega_{3,3} & . & . & . & \omega_{27,3}\\ \omega_{1,4} & \omega_{2,4} & \omega_{3,4} & . & . & . & \omega_{27,4} \end{array}\right)\left[\begin{array}{l} r_{1}\\ r_{2}\\ r_{3}\\ .\\ .\\ .\\ r_{27} \end{array}\right]+\left[\begin{array}{c} b_{2,1}\\ b_{2,2}\\ b_{2,3}\\ b_{2,4} \end{array}\right]\right),$$ (3)

where the weights are denoted as *w* and *ῳ*, *b* and *B* present the biases, *S*(*x*), *I*(*x*), *R*(*x*), and *V*(*x*) are the output performances, while *ϒ* is the activation LSF, which accomplishes the performances between 0 and 1. The activation LSF has a curve of *S*-shaped curve, which presents the nonlinear connotation of inputs and outputs. The mathematical LSF is presented as:


$$\Delta =\frac{1}{1+e^{-x}}$$ (4)

**Fig 1:**
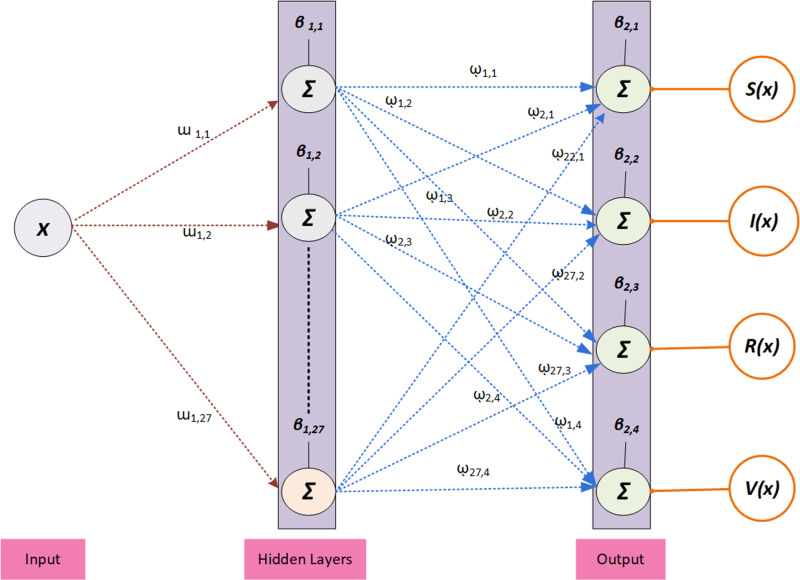
A layer structure for solving the model.

This scheme permits distributions by applying the performances of system parameters, which distribute the difficulty of the neural network in order to examine the probabilistic Bayesian to perform the operative learning. Few diversities for preceding circulations are scale mixture and Gaussian, which contain a number of regularization positions for modifying the system’s complexity. Fig 1 presents the nonlinear mathematical anthrax disease system in animals by the neural outputs and construction. The proposed stochastic computing solver to get the numerical solutions of the nonlinear anthrax disease system in animals is simplified by applying the ‘nftool’ command, while the optimization is executed in the MATLAB software, and other settings are tolerance 10^-08^, 1000 iterations, *n*-folded cross certification, 1/100 step size, twenty-seven neurons, logistic sigmoid function, a feed forward neural construction, and a Bayesian regularization method. Bayesian regularization presents an essential role to estimate the parameters in order to predict the dynamics based on the anthrax disease spread in animal. As mathematical systems are helpful to simulate anthrax transmission in animals, while parameter uncertainty can perform the imprecise predictions. Currently, Bayesian regularization has been used in many applications, some of them are sensorless measurement of pump operational state [27], thermal distortion control in 3D printing [28], predicting the compressive strength of a quaternary blend concrete [29], biomechanical investigations and prediction of lower extremity joint movements [30], and prediction of strength characteristics based on the fly-ash and bottom-ash [31]. Fig 1 shows that the construction of the layers for solving the model.

**Fig 2:**
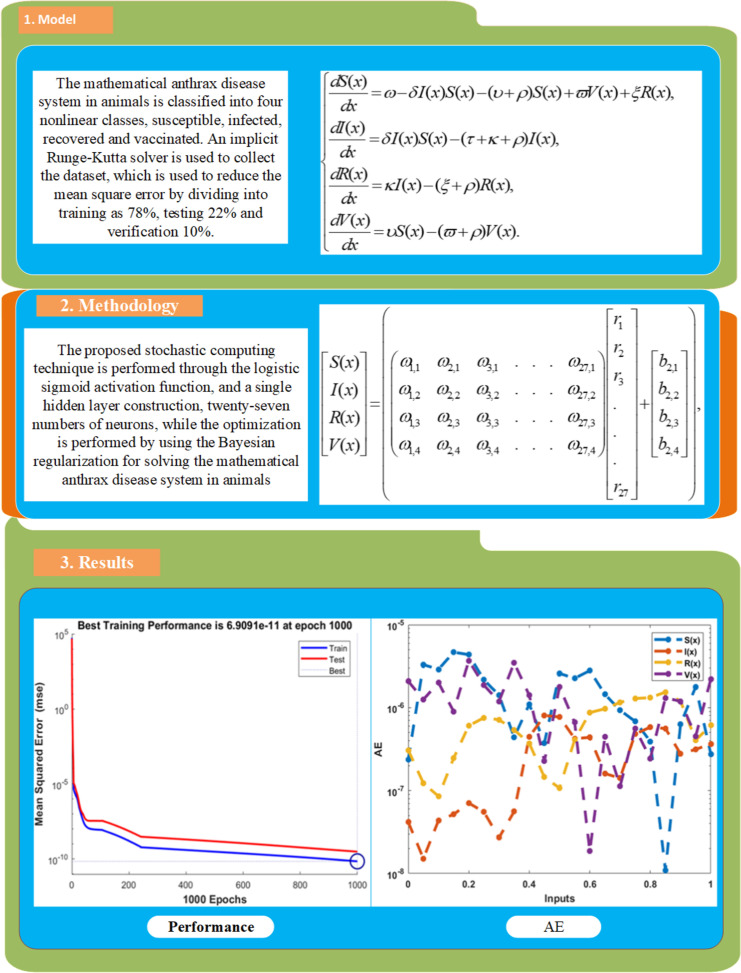
Workflow depictions of the nonlinear system.

Fig 2 illustrates the mathematical anthrax disease model in animals, neural network construction and some graphical illustrations that have been calculated based on the model.

**Fig 3:**
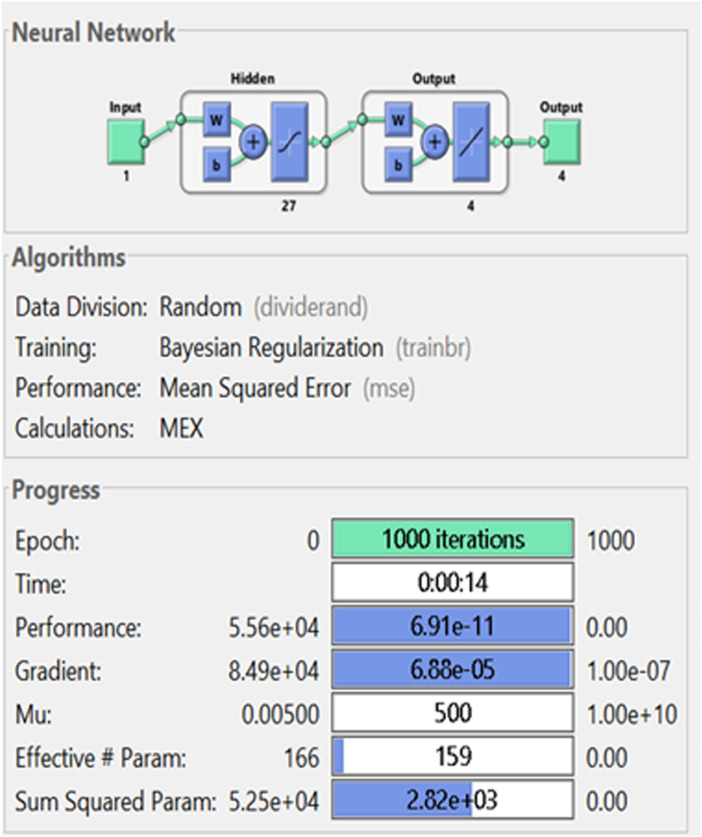
A structure of single hidden layers.

Fig 3 is based on the input, hidden, output layers construction for solving the model by the construction of ANN based Bayesian regularization. Twenty-seven neurons have been used, which shows that the small values of neurons, premature convergence or underfitting is obtained. On the other hand, overfitting or high complexity is achieved by taking large numbers of neurons.

## 4. Discussion of the numerical results

The current section presents the results of anthrax disease model in animals by using the construction of ANN based Bayesian regularization. The appropriate values of the parameter are used in the system (1) as:

**Case 1:** Consider

ω=200, δ=0.0001, υ=0.1, ρ=0.001, ϖ=0.03, ξ=0.02, τ=0.1, and κ=0.01, whereas the ICs are 0.1, 0.2, 0.3 and 0.4 used in model (1) as:


$$\{\begin{array}{l} \frac{dS(x)}{dx}=200-\left(0.0001I(x)-0.101\right)S(x)+0.03V(x)+0.02R(x),S(0)=0.1\\ \frac{dI(x)}{dx}=0.0001I(x)S(x)-0.111I(x),I(0)=0.2\;\\ \frac{dR(x)}{dx}=0.01I(x)-0.021R(x),R(0)=0.3\\ \frac{dV(x)}{dx}=0.1S(x)-0.031V(x),V(0)=0.4 \end{array}$$ (5)

**Case 2:** Consider ω=200, δ=0.0001, υ=0.1, ρ=0.001, ϖ=0.02, ξ=0.02, τ=0.1, and κ=0.01, whereas the ICs are 0.2, 0.3, 0.4 and 0.5 used in model (1) as:


$$\{\begin{array}{l} \frac{dS(x)}{dx}=200-\left(0.0001I(x)-0.101\right)S(x)+0.03V(x)+0.02R(x),S(0)=0.2\\ \frac{dI(x)}{dx}=0.0001I(x)S(x)-0.111I(x),I(0)=0.3\\ \frac{dR(x)}{dx}=0.01I(x)-0.021R(x),R(0)=0.4\\ \frac{dV(x)}{dx}=0.1S(x)-0.031V(x),V(0)=0.5 \end{array}$$ (6)

**Case 3**: Consider ω=200, δ=0.0001, υ=0.1, ρ=0.001, ϖ=0.02, ξ=0.02, τ=0.1, and κ=0.01, whereas the ICs are 0.3, 0.4, 0.5 and 0.6 used in model (1) as:


$$\{\begin{array}{l} \frac{dS(x)}{dx}=200-\left(0.0001I(x)-0.101\right)S(x)+0.03V(x)+0.02R(x),S(0)=0.3\\ \frac{dI(x)}{dx}=0.0001I(x)S(x)-0.111I(x),I(0)=0.4\;\\ \frac{dR(x)}{dx}=0.01I(x)-0.021R(x),R(0)=0.5\\ \frac{dV(x)}{dx}=0.1S(x)-0.031V(x),V(0)=0.6 \end{array}$$ (7)

**Fig 4:**
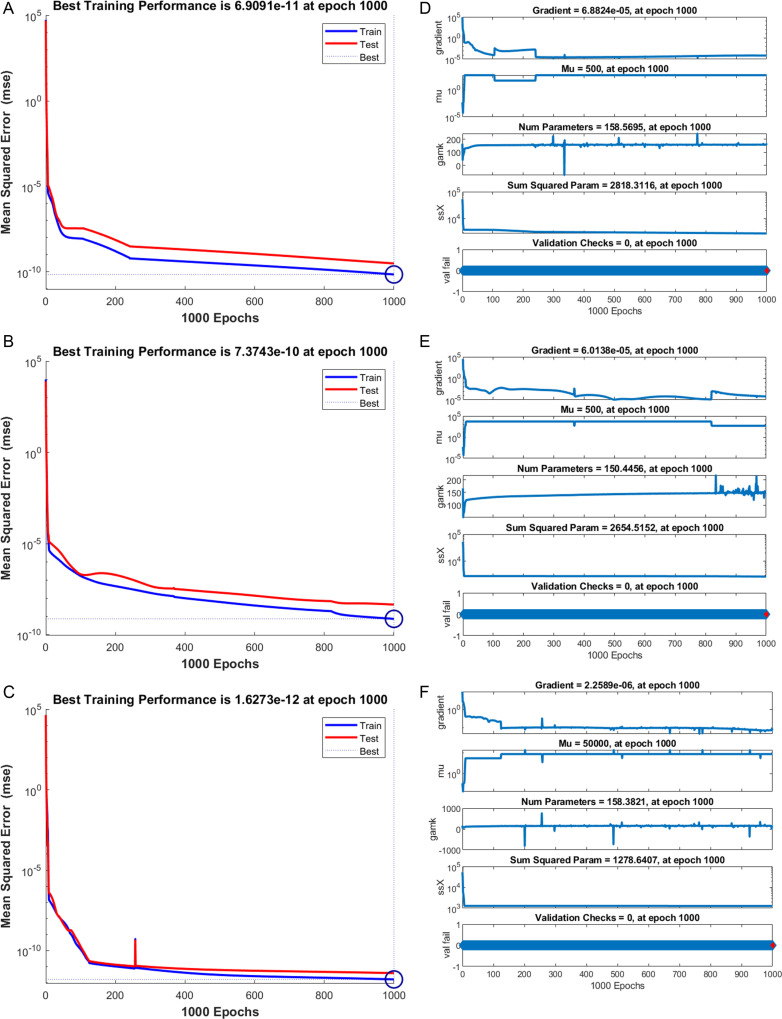
Training and EH for the anthrax disease model in animals. (a) Training (1) , (d) Gradient (1), (b) Training (2), (e) Gradient (2), (c) Training (3), (f) Gradient (3).

**Fig 5:**
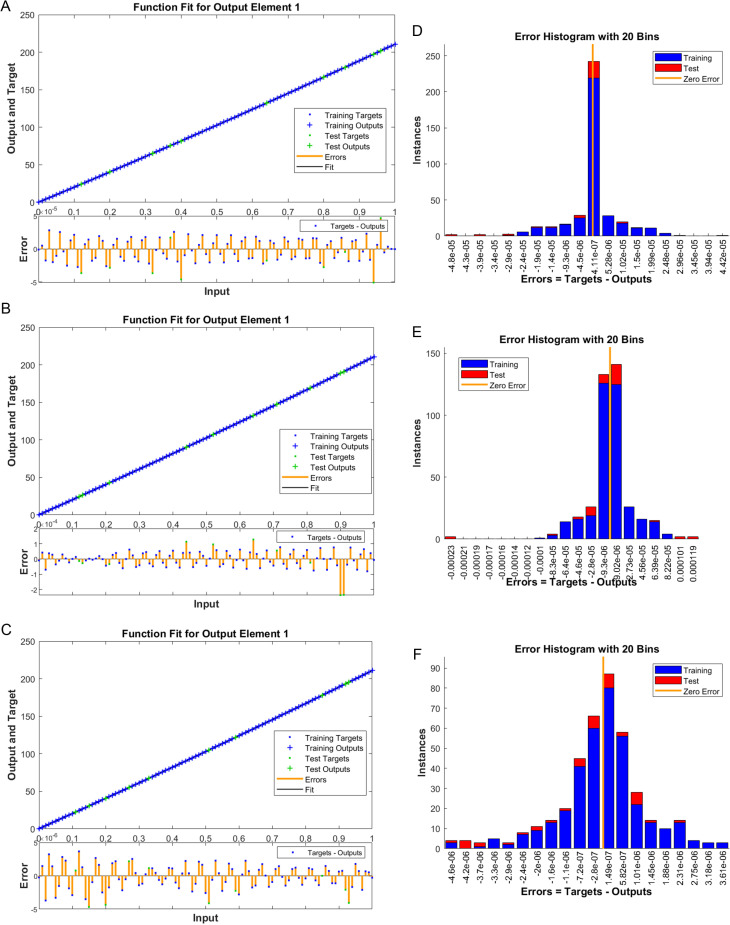
Function fit and EH performances for the anthrax disease model in animals. (a) Function fit (1), (d) EH (1), (b) Function fit (2), (e) EH (2), (c) Function fit (3), (f) EH (3).

**Fig 6:**
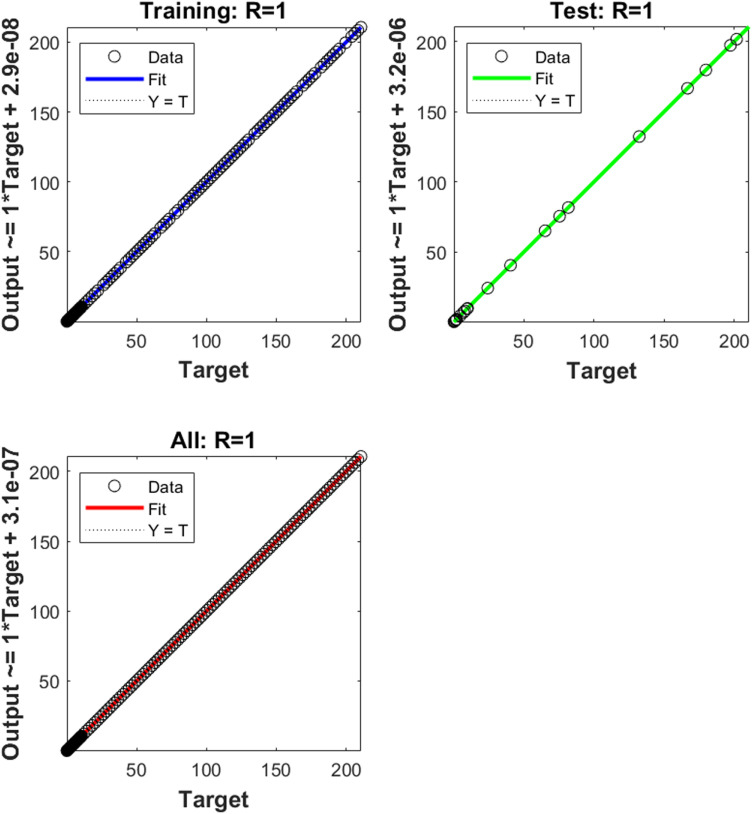
Regression for case (1).

**Fig 7:**
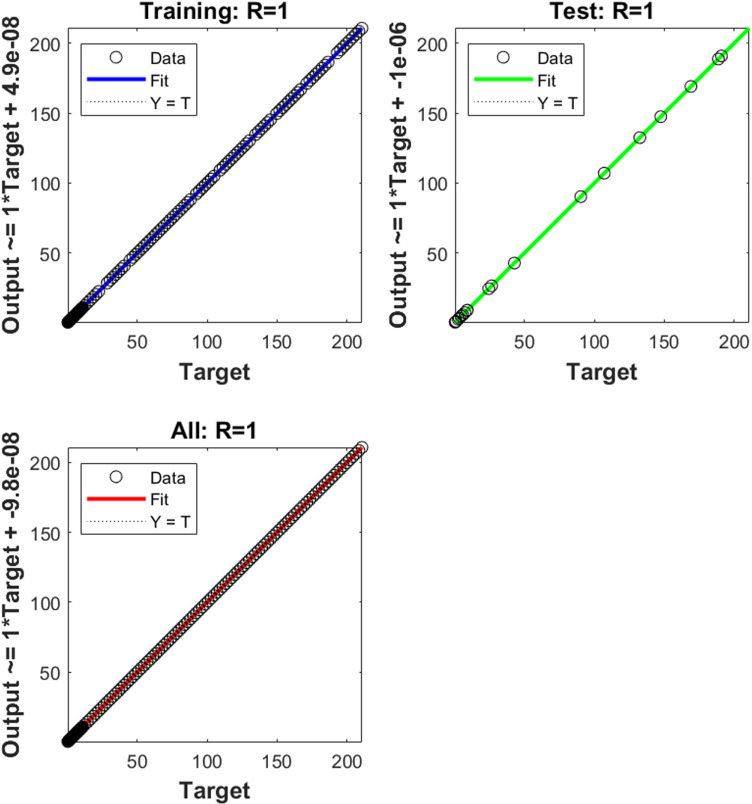
Regression for case (2).

**Fig 8:**
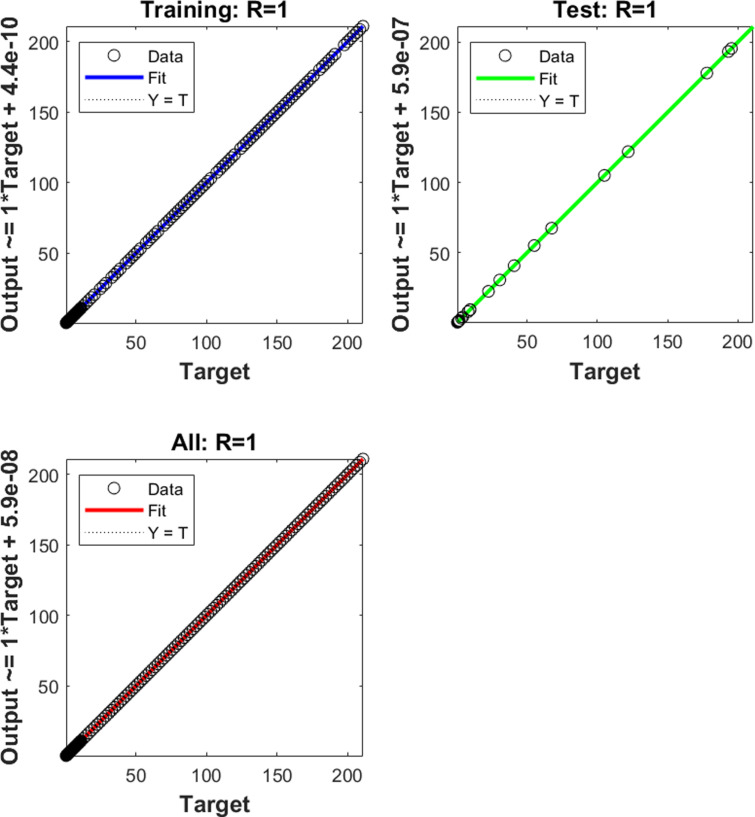
Regression for case (3).

The numerical results of the model using the stochastic ANN based Bayesian regularization are presented in Figs 4 to 8. The optimal substantiation and the transition state (TS) performances are given in Fig 4. The optimal training performances for the anthrax disease in animals using the process of ANN based Bayesian regularization have been presented through the cross-validation procedure. These results are based on the single layer structure, LSF and 27 neurons, respectively. The learning rate is 6.9091 ×  10^-11^, 7.3743 × 10^-10^, and 1.6273 ×  10^-12^ yielded the reduced mean square error (MSE) at epochs 1000 for each case. Generally, the smallest performances of MSE in the structure of neural network is considered a significant step to enhance the scheme’s efficiency, which certificates to indorse the hidden data. TS of the anthrax disease system has been precisely seized by monitoring the gradient 6.8824 ×  10^-05^, 6.0138 × 10^-05^, and 2.2589 ×  10^-06^ for cases 1 to 3, mean update parameter (Mu) is 500 for case 1 and 2 and 50000 for case 3, and numbers of model parameters (Num parameter) are 154.4753, 153.4661, and 155.1755. These gradient measures are extensively applied for solving the model. As the system come up to the TS, the values of gradient decreased, stabilization of Mu is performed, and optimization of Num parameter is validated. These all collective metrics authorize the model’s transition through the process of training to generalization, which enable the consistent predictions based on the anthrax disease dynamics in the populations of animals. Fig 5(a to c) represents the performances of fitting curves of the model. The function fit for the output elements based on the anthrax disease model shows the excellent agreement in the actual and predicted measures. These optimal measures have been experiential by using the outcomes matching for each variation of the model. The metrics based on the function fit confirmed the system’s competence to reliably approximate the dynamics of anthrax disease, which allowing informed decision to form the disease control policies and public health interferences. The performances based on the error histogram (EH) are reported in Fig 5(d to f), which are 4.11 ×  10^-07^, 9.02 ×  10^-06^ and 1.49 ×  10^-07^ for each case of model. In the process of neural network, the metrics based on the EH are applied for numerous applications based on network evaluations, bias corroboration, and error assessment. EH shows a practical scheme that provides platform valuation and optimization by identifying the efficiency of neural network. Figs 6–8 indicate the regression values of the model using the ANN based Bayesian regularization. The regression coefficients for the anthrax disease system present the noteworthy association between disease dynamics and predictor variables. The plots of regression represent the robust preparation through the input ranks to achieve the features of long-term purposes. The coefficient of regression (*R*^*2*^) is 1 for each case, which shows the perfection of the model. Table 1 shows different performances of the solver for the nonlinear anthrax disease model.

**Fig 9:**
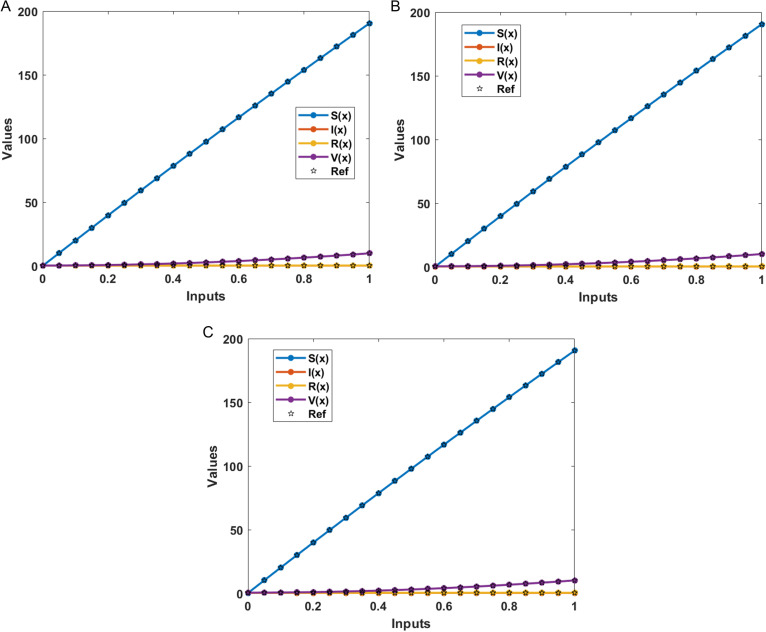
Result matching for the model. (a) Outcomes: Case 1. (b) Outcomes: Case 2. (c) Outcomes: Case 3.

**Fig 10:**
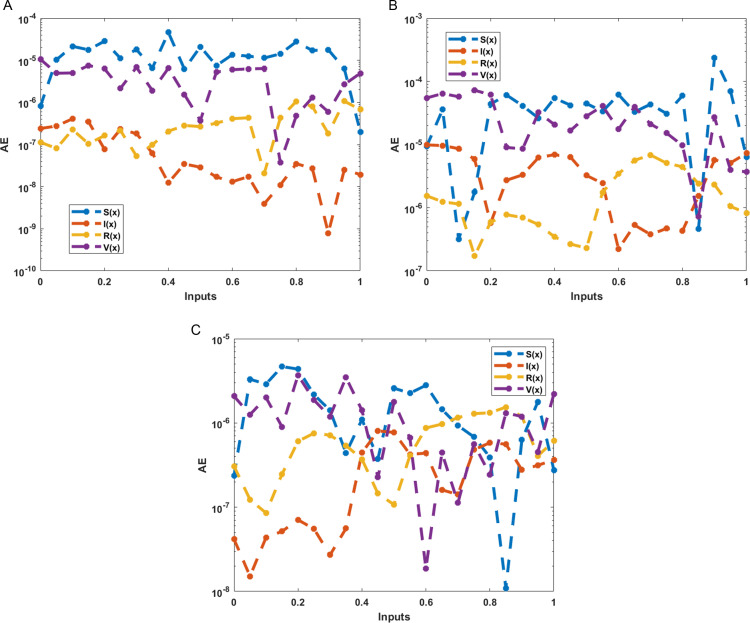
AE performances for the anthrax disease model in animals. (a) AE: Case 1. (b) AE: Case 2. (c) AE: Case 3.

The numerical solutions of the system are illustrated in Fig 9 by the stochastic computing ANN based Bayesian regularization performances. The matching of results for each category of the model present the class-wise accuracy, which represents the exactness of the scheme. The AE performances for each case of the anthrax disease model in animals are presented in Fig 10. AE presents a quantitative measure of the proposed and the reference values, mathematically given as AE=|proposed values – reference performances | . AE shows a straightforward sign of the error’s magnitude, which allow to get easy comparison and performance assessment based on the different systems, prediction schemes and algorithms.

**Table 1:** Different values of the anthrax disease model.

Case	MSE					
**Testing**	**Training**	**Gradient**	**Performance**	**Epoch**	**Time**
I	3.05639 × 10^-10^	6.90914 × 10^-11^	6.88 × 10^-05^	6.91 × 10^-11^	1000	14 Sec
2	4.47464 × 10^-09^	7.37432 × 10^-10^	6.01 × 10^-05^	7.37 × 10^-10^	1000	17 Sec
3	4.00705 × 10^-12^	1.62728 × 10^-12^	2.26 × 10^-06^	1.63 × 10^-12^	1000	12 Sec

The software for the neural network is used MATLAB R2019a, while the dataset is calculated in Mathematica13.3 software.

## 5. Conclusion

This research shows the numerical presentations of the anthrax disease model in animals by applying a machine learning stochastic procedure. The mathematical anthrax disease system in animals is classified into four groups, susceptible, infected, recovered and vaccinated. Some of the comments are presented as:

- The numerical values of the mathematical anthrax disease model in animals have been presented effectively by designing a machine learning stochastic procedure.
- The proposed stochastic solver has been applied successfully to deal the nonlinearity of the mathematical model.
- An implicit Runge-Kutta solver has been implemented to collect the dataset, which is used to reduce the MSE by dividing into training as 78%, testing 12% and verification 10%.
- The proposed stochastic computing technique has been performed through the logistic sigmoid activation function.
- A single hidden layer construction along twenty-seven numbers of neurons has been applied to solve the model.
- A Bayesian regularization has been used in the process of optimization for the mathematical model.
- The designed procedure’s correctness is authenticated through the overlapping of the results.
- The negligible AE around 10^-06^ to 10^-08^ shows the correctness of the solver.
- The best training performances are calculated around 10^-10^ to 10^-12^ for cases 1 to 3 of the model.
- The regression coefficient values have been performed as 1, which shows the perfection of the mathematical model.
- Some tests based on the error histogram, and state transition values enhance the reliability of the proposed stochastic machine learning approach.

In future, the proposed stochastic ANN based Bayesian regularization solver can be implemented to solve several nonlinear applications, like singular systems, fluid dynamics, and biological systems.
